# Accuracy of Ultrasound for Fatty Liver Assessment

**DOI:** 10.1016/j.ajmo.2026.100144

**Published:** 2026-09-30

**Authors:** Erica W. Chen, Thayse Lozovoy Madsen-Barbosa, Elisha Poddar, Azade Tabaie, Radhika Vij, David S. Weisman

**Affiliations:** a Georgetown University School of Medicine, Washington, DC; b Department of Medicine, MedStar Union Memorial Hospital, Baltimore, MD; c Center for Biostatistics, Informatics and Data Science, MedStar Health Research Institute, Hyattsville, MD

**Keywords:** Fatty liver, MASLD, POCUS, Point of care ultrasound, Primary care, Ultrasound Fatty Liver Index (US-FLI)

## Abstract

**Background:**

Metabolic dysfunction-associated steatotic liver disease (MASLD) is increasingly prevalent, particularly among individuals with obesity and Type 2 diabetes. Early identification of MASLD is critical, yet access to formal imaging is often limited in primary care. Point-of-care ultrasound (POCUS) with the Ultrasound Fatty Liver Index (US-FLI), a semi-quantitative scoring tool, has demonstrated strong diagnostic accuracy but remains underutilized and is rarely included in standard POCUS training. This pilot study aimed to evaluate the diagnostic performance of US-FLI when performed by a novice POCUS user to explore its potential role as a screening tool for MASLD.

**Methods:**

In a prospective convenience cohort (*n* = 65), US-FLI scores from a novice POCUS user were compared to radiologist-interpreted abdominal imaging. Sensitivity, specificity, positive predictive value, negative predictive value (NPV), and area under the receiver operating characteristic curve were calculated.

**Results:**

Fifteen patients (23%) had confirmed fatty liver disease on standard imaging. The novice user achieved a sensitivity of 0.87, specificity of 0.32, positive predictive value of 0.28, and NPV of 0.89. The area under the receiver operating characteristic curve was 0.61 (95% CI: 0.54-0.67). Raising the US-FLI threshold improved specificity but lowered sensitivity: ≥3 balanced sensitivity (0.73) and specificity (0.62), whereas ≥5 maximized specificity (0.96) at the expense of reduced sensitivity (0.27).

**Conclusions:**

POCUS using US-FLI may serve as a rapid, noninvasive screening approach with high sensitivity and NPV, suggesting potential utility for ruling out MASLD in primary care settings. Larger studies in more diverse patient populations are needed to further validate these findings.

## Introduction

Metabolic dysfunction-associated steatotic liver disease (MASLD), previously termed nonalcoholic fatty liver disease, is the most common cause of chronic liver disease globally, affecting over 25% of the adult population.^1^, ^2^, ^3^ MASLD encompasses a spectrum of liver pathology—from simple steatosis to the inflammation and hepatocellular injury characteristic of metabolic-associated steatohepatitis—driven by dysregulated glucose and lipid metabolism rather than significant alcohol intake.^4^ Advanced progression of MASLD leads to cirrhosis, hepatocellular carcinoma, and liver-related mortality.^5^ In the United States, MASLD currently affects nearly 40% of the population, marking a 50% increase over the past three decades.^6^

The growing prevalence of MASLD parallels rising rates of obesity and Type 2 diabetes, with an estimated 55%-70% of individuals with Type 2 diabetes affected by MASLD.^7^^,^^8^ Type 2 diabetes is also a key predictor of morbidity and mortality in MASLD, and even early-stage disease in this population is associated with an elevated risk of cardiovascular disease and all-cause mortality.^9^^,^^10^

Given the asymptomatic nature of early MASLD, the American Diabetes Association now recommends screening individuals with diabetes or prediabetes—particularly those with obesity—for MASLD.^11^ However, despite the rising prevalence and clinical impact, there are currently no formal guidelines from the United States Preventive Services Task Force or major liver societies endorsing population-wide screening. Early identification of MASLD is critical not only for preventing hepatic complications but also for reducing the growing healthcare and societal costs associated with advanced disease.^12^, ^13^, ^14^ There is a pressing need for a practical, noninvasive screening tool to identify patients at risk, initiate earlier interventions, and reduce the incidence of adverse cardiovascular and hepatic outcomes.

Point-of-care ultrasound (POCUS) offers a practical and accessible option for frontline MASLD screening, especially in primary care and resource-limited settings. The Ultrasound Fatty Liver Index (US-FLI) has previously been shown to have excellent reproducibility with strong discriminative ability across different steatosis grades when compared to the clinical FLI.^15^^,^^16^ A recent meta-analysis by Hernandez et al^17^ demonstrated that POCUS with US-FLI achieved 93% sensitivity and 98% specificity for MASLD detection compared to formal US. Despite this, US-FLI remains underutilized and is not included in standard POCUS training.

In this pilot study, we aimed to evaluate the diagnostic performance of US-FLI when performed by a novice POCUS user, comparing findings to formal imaging. We hypothesized that a novice user would demonstrate adequate diagnostic performance to support further evaluation of US-FLI-guided POCUS as a potential screening tool for MASLD in primary care.

## Materials and Methods

### Patients

This study utilized a prospective convenience sample of adults presenting to the Adult Medicine Center at MedStar Union Memorial Hospital in Baltimore, Maryland. The MedStar Institutional Review Board approved the study.

Eligible participants were adults (≥18 years) with a BMI ≥ 25 (including all obesity classes), a diagnosis of Type 2 diabetes or prediabetes, and a documented abdominal CT or US performed within the past 12 months. Our inclusion criteria take into consideration new changes in fatty liver nomenclature by The American Association for the Study of Liver Diseases, which now encompasses at-risk individuals who have Type II DM, prediabetes, elevated triglyceride levels, and BMI ≥ 25.^1^

Exclusion criteria included critical illness precluding examination, inability to provide informed consent, patients with known cirrhosis, pregnant patients, prisoners, and non-English-speaking patients.

### US Acquisition and US-FLI Assessment

Abdominal US examinations were performed without prior fasting using a Philips Lumify handheld phased array probe by a single novice operator. The novice user was a second-year medical student without formal US training.

The US-FLI, as described by Ballestri et al^18^ (see Table 1), is a semi-quantitative scoring system for hepatic steatosis based on characteristic sonographic features. The total score ranges from 0 to 8, with the presence of liver–kidney contrast serving as the essential diagnostic criterion for steatosis. Additional features include posterior beam attenuation, vessel blurring, poor visualization of the gallbladder wall or diaphragm, and focal fat sparing. Focal sparing is defined as the presence of a focal hypoechoic area adjacent to structures such as the gallbladder wall, portal vein, or falciform ligament. Representative POCUS images demonstrating the sonographic features used to grade hepatic steatosis are shown in Figure 1.

**Table 1:** Ultrasound Score of Fatty Liver Index (US-FLI)

Features	Scoring
Liver-kidney contrast	None = 0 Mild/moderate = 2 Severe = 3
Posterior attenuation of the beam	Absence = 0 Presence = 1
Vessel blurring	Absence = 0 Presence = 1
Difficult visualization of gallbladder	Absence = 0 Presence = 1
Difficult visualization of diaphragm	Absence = 0 Presence = 1
Areas of focal sparing (gallbladder wall, portal vein, or falciform ligament)	Absence = 0 Presence = 1

**Figure 1:**
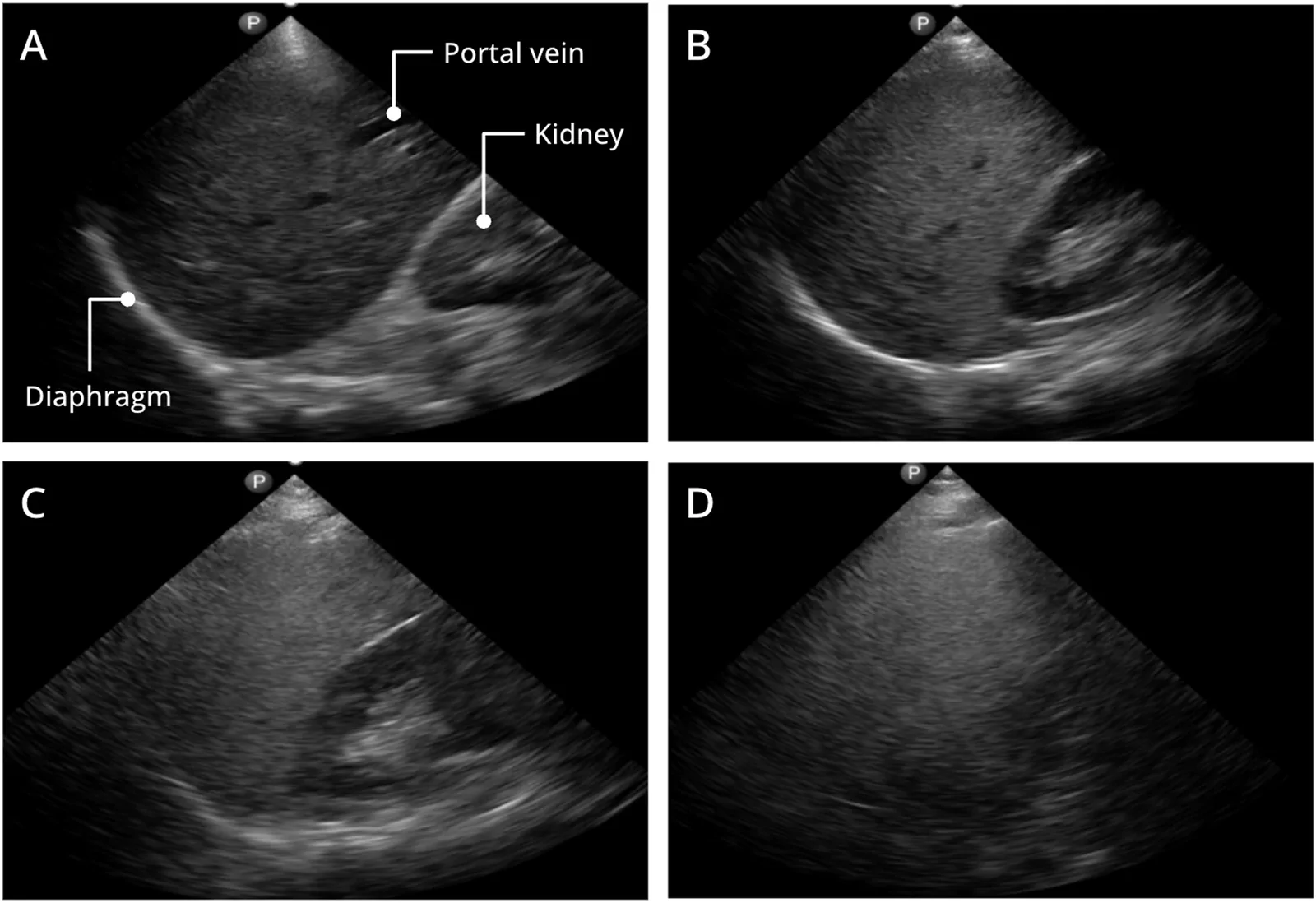
Representative POCUS images of hepatic steatosis. Representative POCUS images of hepatic steatosis. (A) Normal liver: Echogenic portal vein walls are clearly visualized, the diaphragm is well defined, and hepatic echogenicity is comparable to the renal cortex. (B) Mild steatosis: Increased hepatic echogenicity with partial visualization of portal vein walls; the diaphragm remains clearly visualized. (C) Moderate steatosis: Portal vein walls are no longer visualized, the liver parenchyma appears coarser, and only partial visualization of the diaphragm is present. (D) Severe steatosis: Markedly increased echogenicity and posterior attenuation of the ultrasound beam with poor or absent visualization of the diaphragm.

The novice POCUS user had no specific experience or formal training in liver ultrasonography but completed an introductory POCUS course. Before enrollment, the novice performed five supervised practice liver USs and US-FLI assessments under the guidance of the study senior author to standardize image acquisition and scoring technique. The novice user was blinded to clinical data and imaging results during the study.

### Outcome Measures and Statistical Analysis

An enrollment target of 70 patients was set based on feasibility within the student’s dedicated research block. The primary outcome was the diagnostic accuracy of the US-FLI when performed by a novice POCUS user compared to standard imaging, defined as radiologist-interpreted abdominal US or CT scan obtained as part of routine clinical care within 12 months prior to enrollment.

A US-FLI threshold of ≥2 was chosen based on the original US-FLI scoring system developed by Ballestri et al,^18^ in which a score of ≥2 represents the minimum threshold for hepatic steatosis. Diagnostic performance metrics—including sensitivity, specificity, positive predictive value (PPV), and negative predictive value (NPV)—were calculated. Alternative US-FLI thresholds were evaluated in an exploratory post hoc analysis to examine the trade-off between sensitivity and specificity across different cutoffs rather than to identify an optimized diagnostic threshold.

All statistical analyses were performed with R software (version 4.4.1).

## Results

A total of 69 patients were enrolled in the study. Four patients were excluded due to missing data, such as laboratory or imaging results, resulting in a final analytic cohort of 65 patients. Baseline characteristics are summarized in Table 2. The cohort had a mean age of 59.4 years (SD 12.4), with equal median ages across groups (61 years). Females comprised 58.5% (*n* = 38) of the population, and the majority identified as Black (81.5%). Common comorbidities included Type II diabetes mellitus (26.2%), hypertension (58.5%), and coronary artery disease (3.1%). The overall mean BMI was 32.9 kg/m² (SD 5.62). Among the reference imaging studies, 54 patients (84%) had CT imaging, 16 (24%) had abdominal US, and 5 (8%) had both modalities available for review.

**Table 2:** Cohort Characteristics

	No (−MASLD) (*n* = 50)	Yes (+MASLD) (*n* = 15)	Overall (*n* = 65)
Age			
Mean (SD)	59.4 (12.5)	59.7 (12.4)	59.4 (12.4)
Gender			
Female	31 (62.0%)	7 (46.7%)	38 (58.5%)
Race			
Black	42 (84.0%)	11 (73.3%)	53 (81.5%)
White	8 (16.0%)	3 (20.0%)	11 (16.9%)
Asian	0 (0%)	1 (6.7%)	1 (1.5%)
Prediabetes	7 (14.0%)	3 (20.0%)	10 (15.4%)
Diabetes	13 (26.0%)	4 (26.7%)	17 (26.2%)
Hypertension	30 (60.0%)	8 (53.3%)	38 (58.5%)
Hyperlipidemia	1 (2.0%)	0 (0%)	1 (1.5%)
Coronary artery disease	2 (4.0%)	0 (0%)	2 (3.1%)
BMI			
Mean (SD)	32.1 (4.40)	35.7 (8.11)	32.9 (5.62)

MASLD = metabolic dysfunction-associated steatotic liver disease.

### US-FLI Distribution

Analysis of the US-FLI scores revealed that most patients exhibited mild steatosis. Mild fatty liver (US-FLI scores of 2-3) was observed in 44% (*n* = 29). Moderate steatosis (scores of 4-5) was observed in 27% (*n* = 18), while normal findings (scores of 0-1) accounted for 27% (*n* = 18). Notably, no patients were classified as severe fatty steatosis (US-FLI > 5).

### Diagnostic Accuracy of Novice User

The diagnostic accuracy of the novice user was evaluated against radiologist-interpreted abdominal US or CT imaging completed for routine clinical care. Of the 65 patients included, 15 (23%) were confirmed to have fatty liver disease on standard imaging. Compared to this reference, the novice user demonstrated a sensitivity of 0.87, indicating a high ability to detect true positives. However, specificity was 0.32, reflecting a limited ability to rule out false positives. The PPV was 0.28, while the NPV was 0.89.

The overall discriminative performance, as measured by the area under the receiver operating characteristic (ROC) curve, was 0.61 (95% CI: 0.54-0.67) (Figure 2), indicating modest overall discriminative ability with strong sensitivity but with limited positive predictive precision.

**Figure 2:**
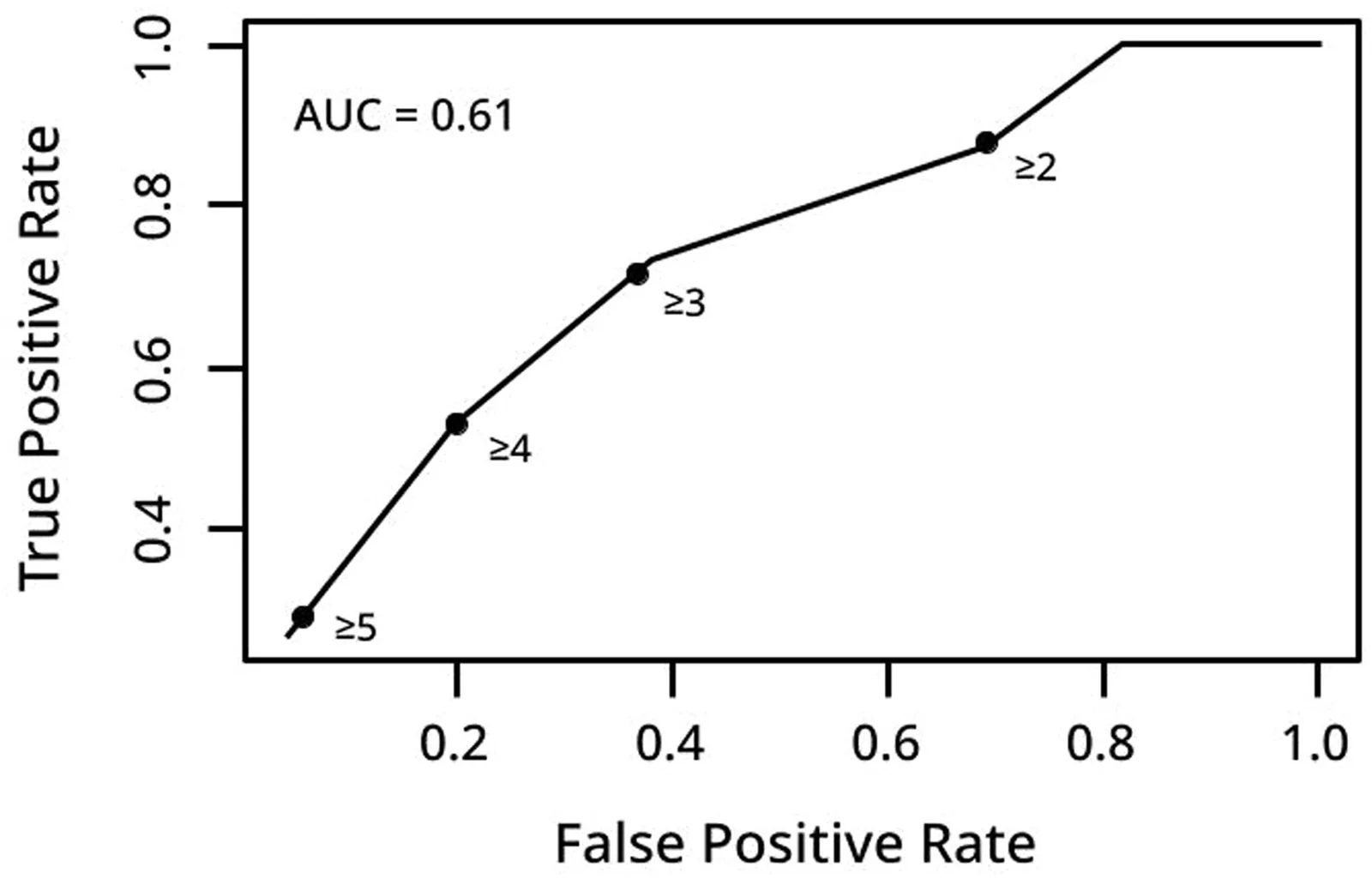
ROC curve associated with novice user. Receiver operating characteristic (ROC) curve for the diagnostic performance of the US-FLI by novice users for detection of MASLD. Points represent selected US-FLI thresholds (≥2, ≥3, ≥4, ≥5) corresponding to sensitivity and specificity values reported in Table 3. AUC = 0.61 (95% CI: 0.54-0.67).

### Effect of Varying US-FLI Cut-Offs

The influence of different US-FLI thresholds on diagnostic performance is summarized in Table 3. As cut-off values increased, sensitivity decreased while specificity improved. A cut-off of 3 provided the most balanced performance for general screening (sensitivity 0.73, specificity 0.62). In contrast, a threshold of 5 maximized specificity (0.96) but significantly reduced sensitivity (0.27), making it more suitable for use as a rule-in tool when confirming disease.

**Table 3:** Assessing the Effect of Different US-FLI Cut-Off Values

US-FLI Cut-Off Values	Sensitivity	Specificity	Positive Predictive Value	Negative Predictive Value
1	1	0.18	0.27	1
2	0.87	0.32	0.28	0.89
3	0.73	0.62	0.37	0.89
4	0.53	0.8	0.44	0.85
5	0.27	0.96	0.67	0.81

## Discussion

MASLD remains an underdiagnosed yet highly prevalent condition with strong associations to cardiometabolic morbidity, particularly among populations with obesity, Type 2 diabetes, and hypertension.^6^^,^^19^ Early identification of MASLD may provide important opportunities for intervention, including lifestyle modification, cardiovascular risk reduction, and appropriate pharmacologic management in patients with more advanced disease. In our urban cohort, risk factors for MASLD were highly prevalent, with a mean BMI in the obese range (32.9 kg/m²), hypertension present in over half of patients, and diabetes in more than a quarter—highlighting the necessity for effective screening tools in primary care.

This pilot study suggests that POCUS combined with the US-FLI may be a feasible method for MASLD screening when performed by a novice user. Compared to expert-standard imaging (abdominal US or CT), the novice user achieved a high sensitivity of 0.87 and a NPV of 0.89, supporting its utility as a rule-out tool. These findings should be interpreted in the context of operator experience; prior studies, such as the systematic review by Hernandez et al,^17^ reported higher diagnostic performance (sensitivity 0.93, specificity 0.98) when POCUS was performed by experienced operators. In our study, specificity was modest (0.32), and the PPV remained low (0.28), suggesting that false-positive results may occur and could lead to downstream consequences such as additional imaging, specialist referrals, increased healthcare costs, and patient anxiety. However, these trade-offs may be acceptable in a screening context where the priority is to identify at-risk individuals while minimizing missed cases and identify patients who warrant further evaluation with formal imaging or additional noninvasive risk stratification rather than a definitive diagnosis. Given its rapid, noninvasive nature and high NPV, US-FLI-guided POCUS may serve as a practical first-line tool for ruling out MASLD in primary care and resource-limited settings.

In this context, US-FLI screening could be integrated with existing noninvasive risk stratification tools such as the Fibrosis-4 (FIB-4) index. FIB-4 is an inexpensive and widely used marker of hepatic fibrosis calculated from age, aspartate aminotransferase, alanine aminotransferase, and platelet count, and is recommended by the American Association for the Study of Liver Diseases for initial risk stratification of patients with MASLD.^20^ Importantly, FIB-4 was designed to detect advanced fibrosis (stage F3-F4) rather than early steatosis, and therefore may have limited sensitivity for early-stage disease.^21^ In this setting, POCUS with US-FLI could serve as an early screening step to identify hepatic steatosis in at-risk patients, followed by fibrosis risk stratification with tools such as FIB-4 to guide referral and further evaluation.

Varying the US-FLI threshold revealed a trade-off between sensitivity and specificity. In our study, a cut-off value of 3 yielded the most balanced diagnostic performance (sensitivity 0.73, specificity 0.62), while a cut-off of 5 prioritized specificity (0.96) at the cost of sensitivity (0.27). Given the limited sample size, this analysis of alternative US-FLI thresholds should be considered exploratory and hypothesis-generating, and requires validation in larger cohorts.

The US-FLI integrates semi-quantitative features such as liver–kidney contrast, posterior attenuation, vessel blurring, diaphragm visibility, gallbladder wall clarity, and focal sparing.^18^ In our study, most patients were classified as mild (44%) or moderate (27%) steatosis, consistent with MASLD’s distributions in general populations.^22^ Some components of US-FLI—such as recognizing posterior beam attenuation or focal fat sparing—were noted by the novice user as more challenging to identify, reinforcing the need for structured training, standardized protocols, and supervised image review to ensure consistent diagnostic reliability. There is currently no national standard for evaluating competency in US-FLI. However, many physicians are now being trained during medical school and residency. We suggest that at least 10-20 supervised evaluations are reasonable.

We acknowledge the limitations of this pilot. The study was conducted at a single center with a relatively small sample size (*n* = 65) using convenience sampling, which introduces a potential selection bias and limits generalizability; interobserver reliability was also not assessed. Additionally, the study population was relatively homogeneous, with a high prevalence of obesity and predominantly a Black patient population, which may limit applicability to other clinical settings. Replication in larger, multi-center cohorts with more diverse patient populations, including individuals across different obesity classes, will be necessary to confirm these findings. Moreover, our reference standard imaging consisted of abdominal US or CT obtained for routine clinical care. Although these modalities are not gold standards for hepatic steatosis detection, such as liver biopsy or Magnetic Resonance Imaging-derived Proton Density Fat Fraction, they are widely used in clinical practice and have high diagnostic accuracy for moderate-to-severe hepatic steatosis, with reported sensitivities approaching the 80%-90% range.^23^ However, their sensitivity declines in mild disease, and imaging performed for nonhepatic indications may not systematically evaluate liver fat, increasing the potential for misclassification when these modalities are used as reference standards. Furthermore, because imaging could occur up to 12 months prior to enrollment, changes in hepatic fat content during that interval may have also affected diagnostic accuracy estimates. Future studies should incorporate standardized prospective imaging to further validate these findings.

This pilot study underscores the feasibility, speed (2-5 minutes per scan), and portability of handheld POCUS devices in the clinic, making them well suited for real-time screening and risk stratification. The noninvasive, low-cost nature of this tool is particularly appealing in primary care, underserved, or resource-constrained settings, where access to formal imaging may be limited. These findings support further investigation of US-FLI-guided POCUS as a pragmatic approach for early MASLD detection, and warrant validation in larger, multi-center studies.

## Conclusion

POCUS utilizing the US-FLI shows promise as a rapid, noninvasive screening tool for MASLD, even in the hands of a novice user. In this pilot study, the observed high sensitivity and strong NPV suggest potential utility as a rule-out tool for fatty liver disease. These findings support the feasibility of integrating handheld US into primary care, especially in settings where access to formal imaging is limited. As MASLD prevalence continues to rise, particularly among populations with obesity and diabetes, scalable and accessible tools such as POCUS may help facilitate earlier identification and enable timely intervention. Further investigation in larger and more diverse patient populations with inter-rater reliability assessments will be important to validate these findings and to define optimal implementation and training strategies for broader clinical use.

## Clinical Significance

• Novice users of handheld POCUS can effectively utilize the US-FLI to exclude MASLD with high sensitivity and strong negative predictive value.
• A US-FLI threshold of ≥3 offers a practical balance between sensitivity and specificity for screening.
• POCUS with US-FLI is a feasible, rapid, and scalable approach to MASLD detection, with strong potential for integration into primary care workflows and resource-limited settings.

## CRediT authorship contribution statement

**Erica W. Chen:** Writing – review & editing, Writing – original draft, Project administration, Methodology, Investigation, Formal analysis, Data curation. **Thayse Lozovoy Madsen-Barbosa:** Writing – review & editing, Writing – original draft, Formal analysis. **Elisha Poddar:** Data curation. **Azade Tabaie:** Software. **Radhika Vij:** Writing – review & editing, Supervision, Project administration. **David S. Weisman:** Writing – review & editing, Validation, Supervision, Project administration, Methodology, Investigation, Funding acquisition, Formal analysis, Data curation, Conceptualization.

## Declaration of competing interest

The authors declare that they have no known competing financial interests or personal relationships that could have appeared to influence the work reported in this paper.
